# Geographical Distribution of Genetic Variants and Lineages of SARS-CoV-2 in Chile

**DOI:** 10.3389/fpubh.2020.562615

**Published:** 2020-09-22

**Authors:** Andrés E. Castillo, Bárbara Parra, Paz Tapia, Jaime Lagos, Loredana Arata, Alejandra Acevedo, Winston Andrade, Gabriel Leal, Carolina Tambley, Patricia Bustos, Rodrigo Fasce, Jorge Fernández

**Affiliations:** ^1^Molecular Genetics Sub Department, Institute of Public Health of Chile, Santiago, Chile; ^2^Section of Respiratory and Exanthematic Viruses, Institute of Public Health of Chile, Santiago, Chile

**Keywords:** COVID-19, SARS-CoV- 2, epidemiology, variants, lineages

## Abstract

The pandemic caused by the new severe acute respiratory syndrome coronavirus 2 (SARS-CoV-2) is a worldwide public health concern. First confined in China and then disseminated widely across Europe and America, SARS-CoV-2 has impacted and moved the scientific community around the world to working in a fast and coordinated way to collect all possible information about this virus and generate new strategies and protocols to try to stop the infection. During March 2020, more than 16,000 full viral genomes have been shared in public databases that allow the construction of genetic landscapes for tracking and monitoring the viral advances over time and study the genomic variations present in geographic regions. In this work, we present the occurrence of genetic variants and lineages of SARS-CoV-2 in Chile during March to April 2020. Complete genome analysis of 141 viral samples from different regions of Chile revealed a predominance of variant D614G like in Europe and the USA and the major presence of lineage B.1. These findings could help take control measures due to the similarity of the viral variants present in Chile, compared with other countries, and monitor the dynamic change of virus variants in the country.

## Introduction

The rapid infection and spread of the new severe acute respiratory syndrome coronavirus 2 (SARS-CoV-2) made it necessary to adopt extraordinary measures, such as quarantines, social distancing, extreme hygienic procedures, and official useful information. In this line, the rapid sharing of trustable information plays a key role in providing guidance to physicians and investigators who advise the authorities to take the best decisions during each stage of the pandemic. Free and quick access to the latest scientific findings contributes to the management and control all aspects of the pandemic, be it scientific or social, without neglecting the quality of this information (1–3).

The outbreak of SARS-CoV-2 was declared as a pandemic on March 11, 2020 by the World Health Organization due to the rapid increase in the number of infected patients outside China (13-fold) and the growing number of countries (up to 113) with cases of coronavirus disease (COVID-19) (4). By August 12, 2020, there are more than 20,162,474 positive cases in 215 countries and territories, with a death toll of 737,417 worldwide (5).

Since the COVID-19 outbreak in China in later December 2019, almost 5 months later and with an unprecedent speed, investigators around the world have uploaded and shared near to 16,000 high coverage genomes, contributing to the development of new diagnostic strategies, tracking the strains for a better understanding of virus spread dynamics and vaccine and treatment development, among other valuable knowledge contributions.

Those genome sequences are hosted in the GISAID Initiative (https://gisaid.org/CoV2020), created to collect influenza viruses information, previous to the SARS-CoV-2 pandemic. To date, there is no official system for naming the phylogenetic diversity, making it confusing and difficult to reach a consensus about strains classification, but there are two ways to group the fast-growing number of isolates, in variants and lineages. According to the guidelines of the GISAID database, the genetic diversity of the isolates was categorized in clades as a consequence of specific single nucleotide polymorphism (SNP) present in the genome. The genetic variants are located in the nucleotide positions 23,403, 26,144, and 28,144 based on the reference sequence NC_045512.2, and the variant's name is represented by a capital letter that corresponds to the amino acid substitution product of the SNP G: Spike—D614G, V: NS3—G251V, S: ORF8—L84S, respectively, and O for other strains that keep some of the nucleotide as the reference strain on that genome position that cannot be assigned to the previous described clades (6). Tracking the cumulative SNPs along the genome has been used to identify the lineages related to the viral spread (7).

RNA viruses are ever-evolving structures, adapting constantly, due to the exposure to variable environments, and the lower viral fitness in these scenarios, for example, interspecies jumps and geographical dissemination, and the high error rates of the RNA-dependent RNA polymerases (RdRp) contribute to fit in the new ambient in just a few generations. The error rates in viral RNA polymerases are near to 10^−4^ compared with 10^−7^-10^−11^ in DNA viruses (8, 9). However, SARS-CoV-2 possesses a non-structural gene with proof-reading activity; thus, its mutation rate is slow, at the pace of 1–2 base substitutions per month across the genome (10).

Up to date, several SNPs across the SARS-CoV-2 genome have been identified (11) in the genes involved in the life cycle of the virus and potential target for antivirals, such as RdRp (12) and Spike protein (13, 14). Until now, there is a few evidence to assign pathogenic or infective special features to the genomic variants (15), but we know that G variant is currently prevalent in the world, with 64% of the sequences found mainly in Europe and North America.

We have already published the phylogenetic analysis of the first four genomes detected in Chile that revealed the two variants derived from strains present mainly in Europe (16).

According to the last report of the Chilean Ministry of Health, until August 9, the cumulative COVID-19 cases had reached 418,196 patients, with 10,402 deaths, where the Metropolitan Region of Santiago concentrates the highest number of affected people, with 69.8% of infected patients and 79% of deaths in the country (17).

In this report, we show the geographical distribution of 141 SARS-CoV-2 isolates collected along the Chilean territory. The complete genome analysis of those samples allows us to identify and classify both genomic variants and lineages.

## Materials and Methods

### Sample Types, RNA Extraction, and Virus Detection

Chilean law by the Supreme Decree 7/2019 mandates the notification of communicable diseases and their surveillance. Throat and nasopharyngeal swab samples were mainly collected. A volume of 140 μl of each sample was used for viral RNA extraction with QIAamp Viral Mini Kit (Qiagen, Cat. No. 52926) in a QIACube extractor. All suspicious cases were confirmed by real-time reverse transcription (RT)-PCR using TaqMan™ 2019-nCoV Assay Kit v1 (Thermo Fisher, Cat. No. A47532).

### Full Viral Genome Amplification

Genome amplification must be performed in two-steps RT and conventional PCR in order to generate a total of 12 fragments around 2.3–2.7 Kbp (16). From total RNA extraction, we performed a first amplification round in order to obtain six cDNA fragments. Each fragment around 5 Kbp was amplified by RT-PCR using 5 μl of RNA, 400 nM of each primer, and the SuperScript® III one-step RT-PCR System with Platinum® Taq Kit (12.5 μl of reaction mix and 0.5 μl of RT/Taq mix, Invitrogen) in a 25 μl final volume. The thermal profile used was 60 min at 45°C, 2 min at 94°C, 40 cycles consisting of denaturation at 94°C, 15 s; annealing at 47°C, 30 s; and elongation at 68°C, 6 min, followed by a final extension for 5 min at 68°C. Each DNA product obtained in the first RT-PCR round is the substrate for the second PCR round, generating two fragments from each first round DNA product. PCR conditions using SapphireAmp fast PCR—hot-start master mix (Takara Bio USA, Cat. No. RR350B) were: initial denaturation for 2 min at 94°C, 30 cycles consisting of denaturation at 94°C, 30 s; annealing at 47°C, 30 s; elongation at 72°C, 1 min; and final extension at 72°C, 5 min.

### Library Generation and Sequencing

The 12 DNA fragments from full genome amplification were pooled, and libraries were prepared with the Nextera XT Library Prep Kit (Illumina, San Diego, CA, USA), purified with Agencourt AMPure XP beads (Beckman Coulter, Brea, CA, USA), and quantified by Victor Nivo Fluorometer (PerkinElmer) using Quant-it dsDNA HS Assay Kit (Invitrogen). The resulting DNA libraries were sequenced on MiSeq (Illumina) using a 300-cycle (total) reagent kit. About 0.3 GB of data was obtained for each sample.

### Phylogenetic and Lineage Analysis

The sequencing quality was analyzed with FastQC software v0.11.8. Readouts were filtered and trimmed using the software BBDuk considering a minimum read length of 36 bases and quality ≥20. Coronavirus assemblies were performed with IRMA software v0.9.3 using as reference NCBI sequence ID NC_045512.2. To identify the G, S, V, and O variants, an alignment was performed using MAFFT v7.458 and Pangolin v1.1.13 package for assigning SARS-CoV-2 genome sequences to global lineage (7).

## Results

### Geographical Distribution of Variants and Lineages

**Figure 1:**
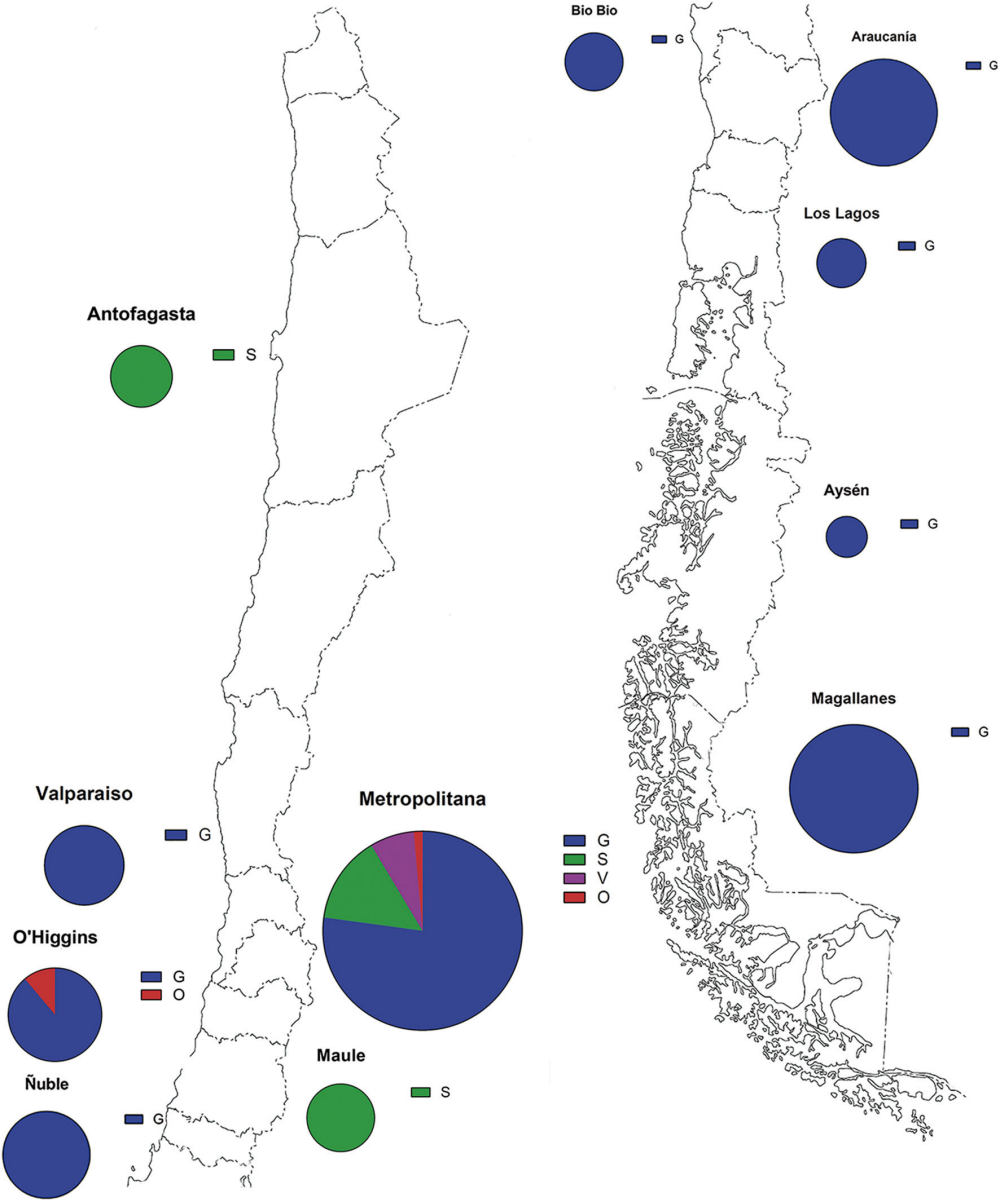
Geographical distribution of the SARS-CoV-2 isolates. Parts of whole graphs represent the proportion of the variants in the regions of Chile. Blue color represents G variant, green S variant, purple V variant, and red O variant. Sphere size is proportional to the number of samples of each zone.

The full genome analysis of 141 SARS-CoV-2 Chilean cases shows a predominance of the variant G in great part of the territory between March 2 and April 5, 2020. The Metropolitan region of Santiago houses near to 8.1 million inhabitants and with 17,979 positive cases represents the most sampled area and the second with incidence rate (165.8) second to Magallanes region (437.9) (7). G variant is widespread over the territory, mostly in central and south regions, such as Valparaiso, Metropolitan, O'Higgins, Ñuble, Bio Bio, Araucanía, Los Lagos, Aysén, and Magallanes. S variants are present in the central region of Maule and also in the northern region of Antofagasta. The less represented variants, V and O, were found in the Metropolitan and O'Higgins regions (Figure 1, Table 1).

**Table 1:** Variants and lineages per geographic region in Chile.

**Region**	**Variant**	**Isolates**	**Lineage**	**Isolates**
Antofagasta	S	2	A.5	2
	G	5	B.1	5
	G	65	B.1	18
			B.1.1	19
			B.1.1.1	3
			B.1.1.10	2
			B.1.5	22
			B.1.5.4	1
	S	10	A.1	1
			A.2	6
			A.5	3
	V	5	B	1
			B.2	2
			B.2.1	1
			B.2.5	1
	O	1	B	1
O'Higgins	G	8	B.1	1
			B.1.1	5
			B.1.5	2
	O	1	B	1
Maule	S	2	A.5	2
Ñuble	G	4	B.1	3
			B.1.5	1
Bio Bio	G	3	B.1	1
			B.1.1	1
			B.1.5	1
Araucanía	G	14	B.1	13
			B.1.1	1
Los Lagos	G	3	B.1	3
Aysén	G	1	B.1	1
Magallanes	G	17	B.1	17
Total		141		141

According to a recent classification criteria (7), lineages of SARS-CoV-2 can be identified by a phylogenetic analysis and grouped by specific SNPs present in the genome. Lineage B is associated with variants G, V, and O, meanwhile lineage A is related to the variant S. The predominant variant G houses the sublineages B.1, B.1.1, B.1.1.1, B.1.1.10, B.1.5, and B.1.5.4, variant V houses the lineages B, B.2, B.2.1, and B.2.5, and variant O is also associated with the lineage B. On the other hand, variant S is associated with sublineages A.1, A.2, and A.5 (Table 1, Supplementary Figure 1). A detailed list of the nucleotide substitutions and genome location is presented in Table 2.

**Table 2:** Nucleotide substitutions associated with viral lineages of Chilean isolates.

**Lineage**		**Position**			**Region of genome**	**Protein product**
A.2	c	8,782	t		ORF1ab-nsp4	Contains transmembrane domain 2 (TM2)
	t	9,477	a		ORF1ab-nsp4	Contains transmembrane domain 2 (TM2)
	c	14,805	t		ORF1ab-nsp12	RNA-dependent RNA polymerase (RdRp)
	g	25,979	t		ORF3a	ORF3a protein
	t	28,144	c		N	Nucleocapsid nucleoprotein
	c	28,657	t		N	Nucleocapsid nucleoprotein
	c	28,863	t		N	Nucleocapsid nucleoprotein
A.5	c	8,782	t		ORF1ab-nsp4	Contains transmembrane domain 2 (TM2)
	c	26,088	t		ORF3a	ORF3a protein
	t	28,144	c		N	Nucleocapsid nucleoprotein
B.1.1	c	241	t		5′UTR	–
	c	3,037	t		ORF1ab-nsp3	Viral protease
	c	14,408	t		ORF1ab-nsp12	RNA-dependent RNA polymerase (RdRp)
	a	23,403	g		S	Spike protein
	g	28,881	a		N	Nucleocapsid nucleoprotein
	g	28,882	a		N	Nucleocapsid nucleoprotein
	g	28,883	c		N	Nucleocapsid nucleoprotein
B.1.5	c	241	t		5′UTR	–
	c	3,037	t		ORF1ab-nsp3	Viral protease
	c	14,408	t		ORF1ab-nsp12	RNA-dependent RNA polymerase (RdRp)
	a	20,268	g		ORF1ab-nsp15	EndoRNase
	a	23,403	g		S	Spike protein
B.1	c	241	t		5′UTR	–
	c	3,037	t		ORF1ab-nsp3	Viral protease
	c	8,389	t	^*^	ORF1ab-nsp3	Viral protease
	c	14,408	t		ORF1ab-nsp12	RNA-dependent RNA polymerase (RdRp)
	a	23,403	g	^**^	S	Spike protein
	g	25,563	t		ORF3a	ORF3a protein

SNPs present in the

* 47.5 and

** *72% of the samples on this lineage*.

### Progression of the Genetic Variants and Lineages in Chile

**Figure 2:**
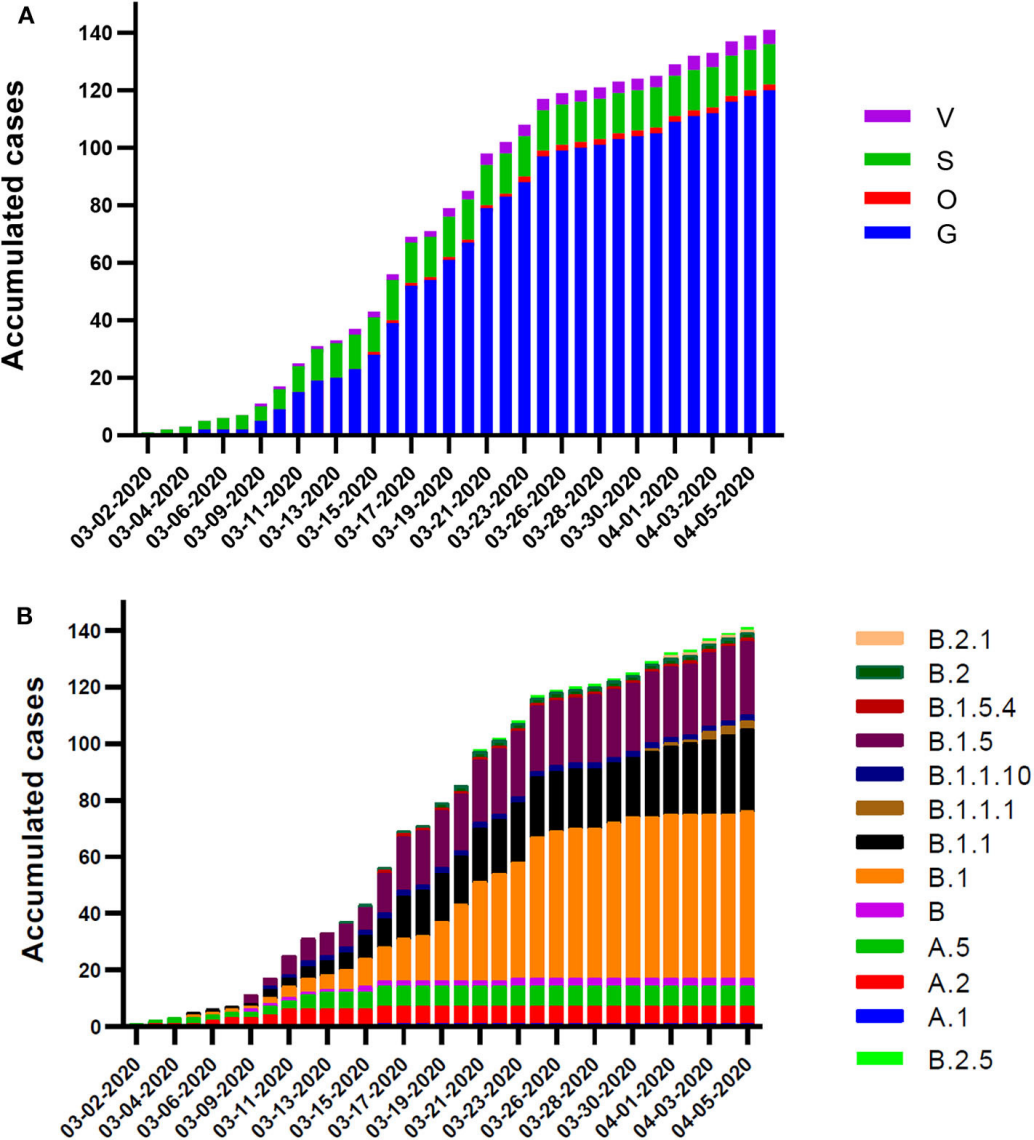
Chronological scheme of the variants and lineages occurrence in Chile. Daily accumulated frequency plot of the sequenced isolates classified according to variant **(A)** and lineage **(B)**. Sampling dates are from March 2 to April 4, 2020.

The different variants and lineages identified in Chilean samples were analyzed and classified according to the isolate date. Tracing of the genetic mutations allows to identify the progression of the introduced events, local contagion, and the emergence of new sublineages by the introduction of new mutations. In a previous report about the first SARS-CoV-2 complete genome analysis, we detected a relative prevalence of variant S over G, a picture of the beginning of the outbreak in Chile in early March 2020. With the progression of the days and more viruses tested, we observed a rapid amount and predominance of G variant over the wild-type genotype, meanwhile the S variant slightly increased on this period. G variant reaches up to 85.1% of the total samples (120 isolates), followed by S with 10% (14 isolates), V with 3.5% (5 isolates), and O with 1.4% (2 isolates) by the beginning of April 2020 (Figure 2A). A similar behavior had the lineage apparition pattern, starting with A.5 (S variant) in early March, and a rapid progression turning predominant sublineages B.1 (41.8%), B.1.1 (20.5%), and B.1.5 (18.4%), all belonging to the G variant (Figure 2B).

## Discussion

In this work, we present the genetic analysis of the SARS-CoV-2 isolates in Chile, the geographical occurrence of Chilean variants, lineages, and tracking of the outbreak. Current data in all fields of investigation regarding variants and lineages are not sufficient to predict infection rates, host susceptibility, or mortality.

At the beginning of the outbreak, Chinese isolates were mainly variant O, the most related to the reference sequence NC_045512.2, but in mid-January 2020, variant S (T 28144 C) and few cases of V (G 26144 T) and G (A 23403 G) started to appear. By the end of March, the variant O reached 58.6% of the sequenced isolates, followed by S, V, and G with 34, 4.8, and 2.7%, respectively. In the rest of the world, variants distribution changes dramatically compared with China, with special focus in Italy and Spain during March and the USA in April. In European countries, the prevalent variant by the end of March was G (Italy 96.1%, Spain 61%), followed by S (Italy 3.9%, Spain 33.3%), meanwhile in the USA, the most representative variant was also G over S (62.9 and 28.6%) by the end of April, according to the uploaded genomes in the GISAID repository.

We also analyze the variant distribution in South America, and it displays a similar behavior to Spain and the USA, showing a prevalence of G variant with 74.3%, followed by S, V, and O variants (16.2, 4.8, and 4.8%, respectively). Complete genome sequences were obtained from the GISAID database from Argentina, Brazil, Chile, Colombia, Costa Rica, Ecuador, Mexico, Panama, Peru, and Uruguay between February 25 and April 18.

Beyond the prevalence of certain variants and lineages in South America and Chile compared with the rest of the world, there is not enough evidence to suggest if a particular phenotype is more or less aggressive than others.

The current number of SARS-CoV-2 complete genomes is growing fast every day, but the disease spread is faster. All the collected information regarding genome sequencing represents <1% of the total infected patients (Chile 0.5%, USA 0.38%, Italy 0.04%, Spain 0.18%, China 0.6%); thus, these epidemiological and phylogenetic studies represent the current picture, and the presented data must be considered as that. In the case of Chile, we are collecting samples in more cities in order to generate a genetic landscape from the entire country. In a previous report about the first cases of SARS-CoV-2 in Chile, we described the introduction of the variants S and G from Southeastern Asia and Europe (16), and most of the current cases belong to the G clade, at the beginning only imported cases, but quickly spread into local transmissions.

The pandemic moved to Europe and America after the China outbreak and followed the complete lockdown of countries. G variant quickly spread across every country it took place, reaching more than 50% of the sequenced samples, except in China where it barely rose up to 2.7%. The variant G looks to be more infectious due to its high prevalence over the other variants in the rest of the world but there is still no conclusive evidence to link a unique SNP with the viral phenotype (15). Many other variables are absolutely necessary to consider, such as effective confinement, ethnic groups, access to quality health services, and vaccination programs, in order to confirm/discard those kinds of assumptions (18–21).

Table 2 shows the positions of the SNPs in the genome that determine the variants and lineages and the ORFs where they are located. The most recurrent locations are in the ORF1ab-nsp3, ORF1ab-nsp4, ORF1ab-nsp12 (RdRp), S, and N. Despite the current information and public knowledge about SARS-CoV-2, it is still not possible to determine the precise effect of the nucleotide mutations and the amino acid substitution in viral infectivity, but it is likely that these mutations are involved in differences in viral pathogenesis.

Success in managing the pandemic does not only depend on how the virus is mutating or winning the race to find effective vaccines and antivirals but also depend on how much we have learned from past viral pandemics, how we develop successful social strategies to stop the spread of the virus, and the way we focus our efforts and resources to generate new knowledge by surveillance and high-quality research (22). In this line, we must keep studying viral phylogeny, epidemiology, and molecular and mathematical modeling, and improve diagnostic, and novel and effective therapies (23).

## Data Availability Statement

The datasets generated for this study can be found in online repositories. The names of the repository/repositories and accession number(s) can be found below: www.gisaid.org, Chile.

## Ethics Statement

Ethical review and approval was not required for the study on human participants in accordance with the local legislation and institutional requirements. Written informed consent for participation was not required for this study in accordance with the national legislation and the institutional requirements.

## Author Contributions

AC participated in the conceptualization, study design, interpretation of data analysis, methodology design, and wrote the whole manuscript. BP participated in methodology design and experimental assays. PT participated in genome assemblies, data analysis, and bioinformatics support. JL and LA contributed to genome sequencing. AA, WA, GL, CT, and PB participated in sample processing and real-time RT-PCR assays. RF participated in the critical review of the manuscript. JF contributed to the conceptualization, study design, and critical review of the content and approved the final version of the manuscript. All authors contributed to the article and approved the submitted version.

## Conflict of Interest

The authors declare that the research was conducted in the absence of any commercial or financial relationships that could be construed as a potential conflict of interest.
