# Supplementary material for: Geographical Distribution of Genetic Variants and Lineages of SARS-CoV-2 in Chile
Source: Front Public Health. 2020 Sep 22;8:562615. doi: 10.3389/fpubh.2020.562615 (PMC7536338; doi:10.3389/fpubh.2020.562615)
Supplement: Supplementary file 1 [file Data_Sheet_1.docx]

**
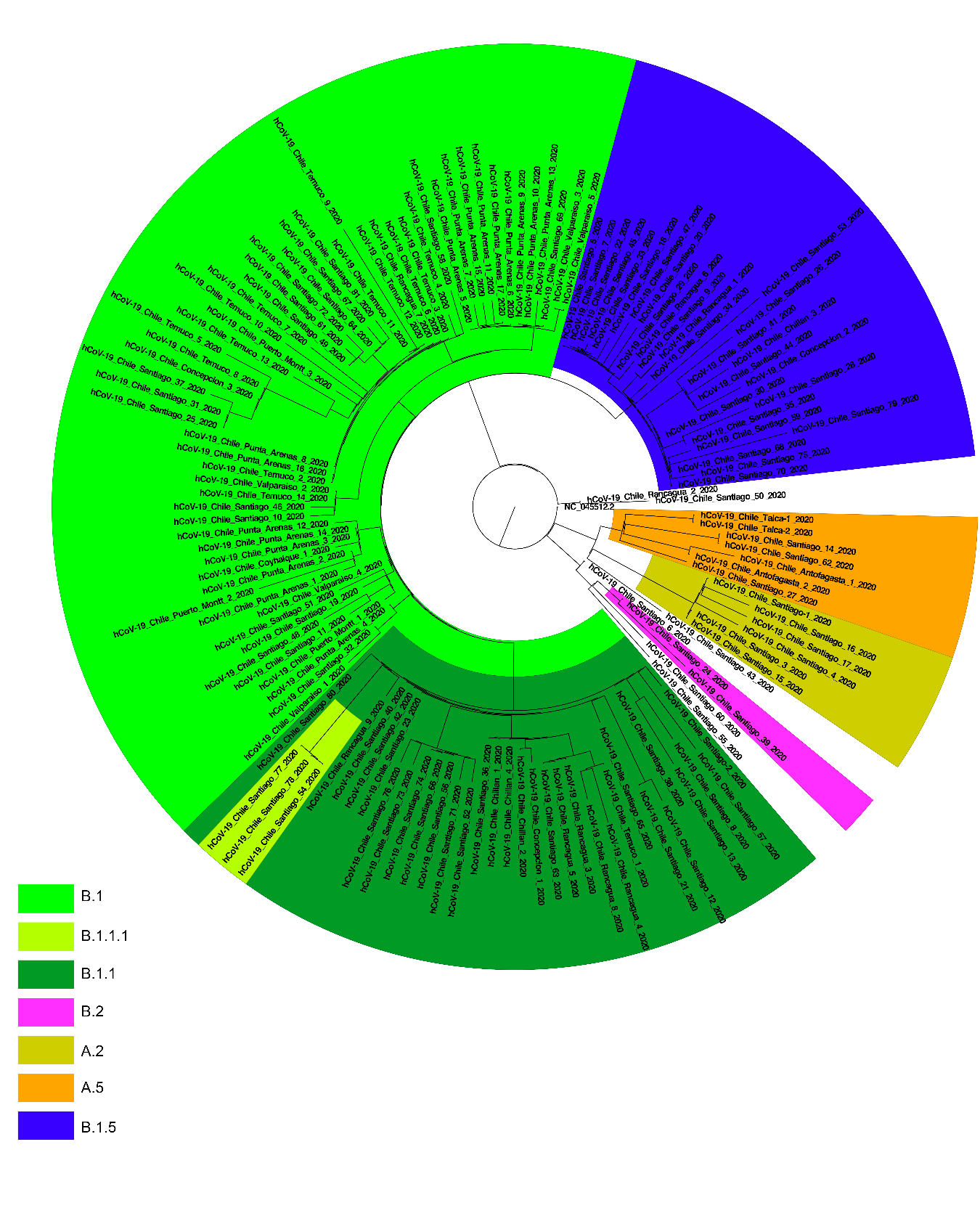
**

**Supplementary Figure 1.** Phylogenetic tree by maximum likelihood generated by IQ-TREE using bootstrapping value of 1000. The most representative active lineages were highlighted by colours.
